# Biohybrid Robotic Jellyfish for Swimming-Enhanced Vertical Ocean Profiling

**DOI:** 10.3390/biomimetics11050325

**Published:** 2026-05-07

**Authors:** Kelsi M. Rutledge, Sean P. Colin, John H. Costello, Noa Yoder, Simon R. Anuszczyk, Kelly R. Sutherland, Brad L. Gemmell, John O. Dabiri

**Affiliations:** 1 Graduate Aerospace Laboratories and Department of Mechanical and Civil Engineering, California Institute of Technology, Pasadena, CA 91125, USA; nyoder@caltech.edu (N.Y.); sanuszcz@caltech.edu (S.R.A.); jodabiri@caltech.edu (J.O.D.); 2 Sensors and Sonar Division, Naval Undersea Warfare Center, Newport, RI 02841, USA; 3 Department of Marine Biology and Environmental Science, Roger Williams University, Bristol, RI 02809, USA; scolin@rwu.edu; 4 Department of Biology, Providence College, Providence, RI 02918, USA; costello@providence.edu; 5 Department of Biology, University of Oregon, Eugene, OR 97403, USA; ksuth@uoregon.edu; 6 Department of Integrative Biology, University of South Florida, Tampa, FL 33620, USA; bgemmell@usf.edu

**Keywords:** biohybrid, jellyfish, ocean sensing, robotics, cyborg, vertical profiling, CTD

## Abstract

Ocean monitoring is essential for understanding climate change and marine ecosystem dynamics, yet achieving comprehensive global coverage remains a challenge in oceanography. Current technologies face limitations in cost, power, hardware, and depth capacity that restrict widespread monitoring capabilities. Here we show that biohybrid robotic jellyfish (*Aurelia aurita*) can serve as autonomous vertical ocean profilers by integrating microcontrollers with positively buoyant sensor payloads, achieving controlled vertical-profiling capabilities. Laboratory experiments demonstrated repeatable up–down trajectories, quantified force balance limits, and identified predictable, size-dependent descent swimming speeds. Field deployments in Massachusetts coastal waters and the open ocean off the Florida Keys demonstrated field operation to ocean depths >25 m with successful in situ temperature and depth measurements. To our knowledge, this represents the first biohybrid jellyfish platform to combine autonomous, pressure-triggered vertical profiling with onboard oceanographic sensing in natural marine environments. This approach leverages the global distribution and remarkable swimming efficiency of living jellyfish while eliminating propulsion power requirements by utilizing the animal’s natural swimming capabilities. While further development is required for long-term ocean deployment, this study lays the groundwork for a new class of biohybrid ocean-sensing platforms with advantages in cost, power, and mission flexibility, providing a pathway toward dense sensor networks and increased ocean monitoring observations.

## 1. Introduction

The vast majority of the ocean remains unexplored, with critical monitoring needs that are becoming increasingly urgent in the face of climate change [1,2,3,4]. Effective ocean monitoring requires comprehensive sampling of Essential Ocean Variables (EOVs) identified by the Global Ocean Observing System (GOOS), including physical parameters (e.g., temperature and salinity), biochemical factors (e.g., oxygen and nutrients), and biological elements (e.g., phytoplankton and zooplankton) [5]. However, the ocean’s vast size, heterogeneity, and spatiotemporal variability present significant challenges for data collection, resulting in many under-sampled or unexplored regions [6,7,8,9,10].

Current ocean monitoring technologies face notable limitations. Profiling floats like the Argo system, one of the largest ocean monitoring networks with over 4000 deployed units [11], revolutionized oceanography by providing unprecedented global coverage of temperature and salinity measurements, yet are typically restricted to depths of 2000 m, with specialized variants reaching 6000 m, and rely solely on passive vertical movement patterns. While Argo floats are substantially cheaper than ship-based measurements, Argo listed the greatest challenge for sustaining a dense, global array has been the high cost ($20,000–120,000 per float). Other technologies, like underwater gliders, offer improved maneuverability over floats, but are generally incapable of station-keeping and are constrained by sensor payload capacity and battery limitations [12,13,14]. Remotely operated vehicles (ROVs) and autonomous underwater vehicles (AUVs) enable more targeted sampling with larger payloads [15,16,17,18,19], but they face challenges including high costs, power constraints [20], and design optimization primarily for horizontal rather than vertical movement [21,22]. These limitations have prompted the development of specialized vertical-profiling vehicles, specifically designed for efficient vertical profiling [9]. Finally, biologging has emerged as an alternative approach, where marine mammals, crustaceans, and fish are equipped with sensors to collect environmental data [23,24,25,26,27,28,29]. This method is relatively inexpensive and provides access to otherwise inaccessible habitats, but it is restricted to the animal’s natural movement patterns [30,31].

Biohybrid robotic control, the ability to steer living organisms along desired trajectories, presents a promising solution that addresses the limitations of both conventional sensing platforms and biologging. Recent advances in biohybrid robotics have demonstrated the feasibility of externally controlling the swimming behavior of moon jellyfish (*Aurelia aurita*) through implanted microelectronics [32,33,34]. This invertebrate species is globally distributed [35], and it offers several inherent advantages as a sensing platform: ubiquitous availability, simple body structure, and remarkable swimming efficiency with the lowest cost-of-transport of any animal [36].

The moon jellyfish possesses a distinctive bell-shaped body with a single muscle layer and eight natural swim pacemakers (rhopalia) that trigger muscle contractions [37]. When a rhopalium activates, it generates a muscle contraction that sets the surrounding water in motion and propels the jellyfish forward (the “power stroke”), followed by a relaxation phase where the bell muscle returns to its uncontracted state (the “recovery stroke”). During recovery, the jellyfish passively recaptures energy from previously created vortices, enabling continued forward propulsion [38,39]. Moon jellyfish are ideally suited for biohybrid control as they do not have a brain, central nervous system, or pain receptors. Hence, jellyfish are not consciously aware of robotic swimming control, and the species studied do not exhibit any detectable stress response associated with the payload attachment. IACUC approval was not required for this study under the institutional and federal animal research guidelines applicable to non-cephalopod invertebrates. The ethical implications of this technology, including invertebrate welfare considerations and responsible development of biohybrid jellyfish systems, have been examined in collaboration with bioethicists; for a detailed discussion, see Xu et al. [40]. The ethical considerations should continue to be evaluated as this technology develops, particularly for future studies involving longer deployments, larger sample sizes, or expanded field use.

Initial biohybrid jellyfish experiments demonstrated the controlled enhancement of swimming capabilities using a small, low-powered microcontroller with two electrodes implanted along the bell margin [33,41]. Through electrical stimulation, biohybrid jellyfish achieved swimming speeds nearly three times faster than their natural locomotion while requiring minimal power only to initiate muscle contraction. Remarkably, this substantial performance enhancement required only a two-fold increase in animal energy expenditure compared to natural swimming, far below the nine-fold increase that proportional scaling would predict [33]. Compared to conventional robots, biohybrid jellyfish require orders of magnitude less external power, consuming between 0.06 ± 0.01 and 0.13 ± 0.03 W kg^−1^, which is 2–3 orders of magnitude less than comparable soft robotic systems while achieving similar swimming speeds [33]. Furthermore, by integrating a 3D-printed mechanical forebody, biohybrid jellyfish achieved swimming speeds up to 4.5 times faster than natural locomotion while also carrying payloads as large as the animal’s own body volume [34].

While prior work established the feasibility of unidirectional control of biohybrid jellyfish through electrical stimulation, these demonstrations lacked the essential components required for practical ocean monitoring, such as an integrated sensor payload and the ability to perform controlled sampling missions comparable to established ocean sampling methods. Thus, the novelty of the present study is the integration of closed-loop depth-based control, positive-buoyancy return-to-surface behavior, and a new sensing payload with onboard temperature sensing to generate repeated vertical oceanographic profiles. This distinguishes the platform from prior biohybrid jellyfish studies focused on locomotor enhancement, as well as biologging studies in which data collection depends on the animal’s natural movement patterns. Here, we demonstrate how biohybrid robot jellyfish could act as operational vertical ocean profilers. We designed a positively buoyant sensor payload that maintains vertical orientation while allowing jellyfish to swim down to a programmed depth and passively float back to the ocean’s surface. These sampling missions mimic the essential profiling function of other ocean sampling floats but with a dramatically reduced cost. We systematically characterize jellyfish swimming performance across a range of body sizes in controlled laboratory settings to identify the relationship between jellyfish size, swimming speed, and vertical-profiling capabilities. This biohybrid platform was tested across three individuals performing 190 vertical profiles totaling 18 h of swimming. Field validation in two distinct marine environments (Woods Hole, Massachusetts and the Florida Keys) demonstrated successful operation under varying ocean conditions while collecting environmental data, confirming the future potential of this platform to function as a practical measurement tool in real-world deployment scenarios.

This work represents the first demonstration of a biohybrid robotic system successfully collecting oceanographic data in natural marine environments. By enabling jellyfish to perform repeated vertical-profiling missions with an integrated sensor payload, we present a new ocean-sensing approach that complements existing technologies through several distinct advantages: global organism availability, minimal power consumption, low manufacturing costs, and the potential for high-density deployment. With additional future development to extend mission duration and enable at-sea data telemetry, this platform could function as a sustained ocean monitoring system. Biohybrid robot jellyfish could be deployed in dense networks of oceanographic sensors in regions previously unmonitored due to technological barriers or economic constraints. This innovative approach represents a critical step toward addressing the need for comprehensive ocean monitoring in the face of accelerating climate change, enabling the collection of Essential Ocean Variables at unprecedented spatiotemporal scales.

## 2. Materials and Methods

### 2.1. Payload Design, Programming, Buoyancy and Energy Calculation

The biohybrid jellyfish payload was designed as two components: a positively buoyant electronics housing and a weighted ballast, connected by a mounting rod. The payload was designed in SolidWorks 2023 and fabricated using a FormLabs (Somervile, MA, USA) 3D printer with orange resin printed at a 100 μm layer height. The hexagonal housing contained the microelectronics payload: a TinyLily microprocessor (TinyCircuits, Akron, OH, USA), MicroSD card reader, buck-boost converter, 250 mAh lithium-ion battery (GM602025-PCB; PowerStream; Orem, UT, USA), 14 bar pressure sensor (MS580314BA01-00; TE Connectivity; Galway, Ireland), and a temperature sensor (TMP119; Texas Instruments, Waltham, MA, USA) with 0.0078 C resolution and ±0.08 C accuracy. The housing was sealed using a double O-ring design to ensure waterproofing at operational depths. The ballast component was fabricated as a solid 3D-printed hemisphere. A threaded mounting rod (0.8 mm diameter) connected the ballast to the electronics housing, passing through the thin, central region of the jellyfish’s stomach. Silver-coated wires (203 μm diameter) were used for electrical stimulation, providing improved durability compared to previous designs. An estimated bill of materials for the consumed payload components is provided in Supplementary Table S1. The estimated per-platform component cost was $48.81, excluding reusable tools and one-time fabrication supplies. Bulk materials including 3D-printed resin, O-rings, epoxy, wire, electrode material, connectors, capacitors, and miscellaneous hardware, were prorated based on the amount used per platform.

The microcontroller was programmed using Arduino IDE 2.3.2 to implement an autonomous vertical-profiling mission. The system used I2C communication to interface with a 14 bar pressure sensor and a temperature sensor. Sensor data was logged to an SD card at 1 Hz, recording timestamps, pressure measurements, and temperature readings. The control algorithm implemented a state-based approach with two primary states: STIMULATING and RESTING. Transitions between states were governed by pressure thresholds, with stimulation beginning at 1110 mbar (a few inches below the surface) and ceasing at 1610 mbar (approximately 1.5 m depth) for laboratory experiments. During stimulation, electrical pulses were delivered at 0.50 Hz with an 8 ms pulse duration. The system was programmed to autonomously detect return to the surface and initiate new dive cycles, enabling continuous vertical profiling with minimal power consumption.

Buoyancy calculations were performed using

*F_b_* = *ρ* × *g* × *V*;

where F_b_ is the buoyant force, *ρ* is the density of seawater (1025 kg m^−3^), *g* is gravitational acceleration (9.81 m s^−2^), and *V* is the volume of water displaced by the object.

The volume of each component was determined using SolidWorks and verified through water displacement measurements. The total displaced volume of the entire payload was 128.0 cm^3^ with a total mass of 124.93 g, generating a buoyant force of 1.287 N and a weight force of 1.226 N. The net buoyancy of the total payload was 0.061 ± 0.003 N. This small positive buoyancy ensured that the biohybrid system would return to the surface when not actively swimming, while still being sufficiently small to allow the jellyfish to overcome it during electrically stimulated swimming at 0.50 Hz. The error range was determined through repeated measurements of component masses (±0.1 g) and volumes (±0.2 cm^3^).

The energy consumption of the platform was quantified by recording the battery’s voltage drop over time and converting the observed voltage to charge, then energy and power. A single-cell lithium-ion battery (nominal 3.7 V, Cnom = 250 mAh) powers the biohybrid platform. We recorded the initial voltage (V_start_), final voltage (V_end_) and voltage drop (ΔV) while the electrodes were stimulating (0.5 Hz) for three time intervals (t). Assuming a linear state-of-charge relation across the 4.20 → 3.00 V usable window (ΔV_use_ = 1.20 V), we measured the capacity consumed using

$$\frac{\Delta C}{C_{nom}}=\frac{\Delta V}{{\Delta V}_{use}}$$

This linear approximation is supported by the relatively flat voltage state-of-charge profile observed in lithium polymer batteries over the nominal discharge range, which allows for the proportional estimation of capacity consumption from voltage drop [42,43].

The energy consumed was measured using

$$E=\Delta CV_{avg};$$

$${\;V}_{avg}=\frac{1}{2}{(V}_{start}+V_{end})$$

The instantaneous power was measured using

$$P=\frac{E}{t}$$

The mass-specific power was obtained by dividing P by the mass of the payload. The cycle-averaged power was calculated by multiplying P by the duty cycle of active swimming, determined from the ratio of powered-descent time to total vertical-profile time (~20% of time spent profiling is active swimming).

### 2.2. Vertical Profiling in Laboratory

Laboratory vertical-profiling experiments were conducted to characterize the swimming performance of biohybrid jellyfish across different body sizes (Video S1). Three *Aurelia aurita* individuals of varying sizes (15.2 cm diameter, 17.8 cm diameter, and 18.5 cm diameter) were individually tested in a vertical acrylic tank of artificial seawater made from instant ocean mixed at 34–35 ppt. Each jellyfish was tagged with colored elastomer markers for individual identification. Jellyfish were tested on 3 separate days for 2 h swimming durations, with 2 days between each testing day.

To prevent unintended swimming during the experiments, jellyfish were placed in 5-gallon buckets with 3 gallons of seawater overnight, which temporarily reduced the frequency of their natural pulsing behavior. Jellyfish were programmed to swim until they reached a depth of approximately 1.5 m, after which stimulation ceased, and the jellyfish floated passively back to the surface. The experiments resulted in a total of 190 vertical profiles across 18 h of swimming (n = 53, 67, and 70 profiles for the small, medium, and large jellyfish, respectively). Swimming behavior was recorded using a GoPro HERO12 camera at 30 frames per second.

Video analysis was performed using a custom semi-automatic MATLAB 2024b tracking algorithm (Video S2). Before processing, 2 h swimming videos were down-sampled to every 10th frame. A total of 30 reference keyframes were manually labeled on videos to establish reliable jellyfish positions. Between these keyframes, a brightness-weighted centroid detection method was used to track the jellyfish within an adaptive search window (150–250 pixels). The algorithm applied background subtraction and contrast enhancement to isolate the jellyfish from the tank. Then, it identified the weighted centroid of bright pixels (exceeding a 0.7 threshold) within the search region to precisely locate the jellyfish. The search window dynamically adjusted based on jellyfish movement, while linear interpolation between keyframes provided initial position estimates that were subsequently refined by centroid detection. This approach yielded accurate frame-by-frame vertical positioning data for detailed swimming performance.

### 2.3. Vertical Profiling in Field

#### 2.3.1. Marine Biological Laboratory

Field testing of the biohybrid jellyfish vertical-profiling system was conducted at the Marine Biological Laboratory (MBL) on 9 August 2025, at a coastal site 6 m deep near Woods Hole Waterfront Park (41°31′29.1′′ N 70°40′23.8′′ W; Video S3). Ocean conditions during testing were challenging, with data from the WHOI weather station (Station BZBM3) and Nantucket Sound buoy (Station 44020) showing high wind and wave activity (Video S4). Stations recorded wind speeds of 9.0 m s^−1^ (18 knots) and wave heights of 0.67 m (2.2 feet) with a dominant period of 3.33 s, resulting in visible whitecaps. Jellyfish were equipped with the sensor payloads on the MBL dock and passed down to divers in the water in Ziploc bags. Divers released the biohybrid jellyfish into the water and followed the platform while it completed vertical profiles, taking video footage. There was vertical drift at the field site, and divers and jellies drifted in the horizontal direction while completing vertical profiles.

#### 2.3.2. Keys Marine Laboratory

Additional field testing was conducted at the Keys Marine Laboratory (KML) in Layton, FL, USA (24°49′44.4′′ N 80°49′04.8′′ W), with the primary objective of evaluating the biohybrid platform’s performance at greater depths and confirming sensor performance. These trials specifically aimed to test the system’s capabilities at depths up to 25 m while incorporating a handheld SonTek CastAway-CTD (YSI, Yellow Springs, OH, USA) for precise ocean temperature measurements to compare to the onboard biohybrid jellyfish temperature sensor. Testing was carried out via boat deployments at two designated sites: a shallow location (6 m depth) and a deeper-water site (~27 m depth) in the open ocean a few miles offshore of KML.

## 3. Results and Discussion

### 3.1. Biohybrid Jellyfish Profiling System Design

**Figure 1:**
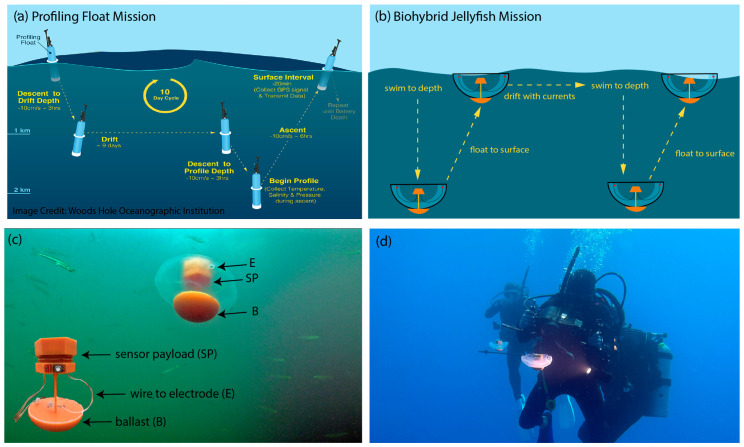
Biohybrid robot jellyfish as buoyancy-controlled vertical-profiling platforms. (**a**) Conventional profiling float mission showing the “park-and-profile” paradigm where floats descend to drift depth, collect data at predetermined intervals, and return to surface to transmit data. (**b**) Biohybrid jellyfish mission design mimicking the profiling float approach, with jellyfish actively swimming downward against positive buoyancy and passively floating back to the surface, creating an autonomously repeating vertical profiler. (**c**) Photograph of biohybrid robot jellyfish integrated with its associated payload (sensor payload, electrodes, and ballast) vertically profiling in shallow coastal waters off Woods Hole Waterfront Park, MA, USA. (**d**) Photograph of two biohybrid robot jellyfish vertically profiling while divers film the descent in the open ocean several miles offshore in the Florida Keys.

The present biohybrid approach transforms living jellyfish into autonomous vertical-profiling platforms through the integration of a custom-designed payload and control system (Figure 1). The integrated payload combines an electronics housing with an oceanic temperature sensor and a counterbalancing ballast, achieving a slight positive buoyancy (0.061 ± 0.003 N) that enables the platform to return passively to the surface after active descent to a preprogrammed depth monitored by an onboard pressure sensor. This design creates an autonomously repeating vertical profiler that alternates between electrically stimulated downward swimming and passive upward drift, collecting oceanographic temperature data throughout the water column.

This operational paradigm is inspired by the “park-and-profile” mission established by Argo floats, with key adaptations for the biohybrid platform. As shown in Figure 1a, conventional profiling floats descend to a drift depth, collect data while drifting, then descend further to begin their profile before returning to the surface to transmit findings. In contrast, the biohybrid jellyfish (Figure 1) currently employ a simplified vertical sampling approach, swimming down to a target depth and floating back to the surface while gathering data. By modifying the current pressure sensor controls, biohybrid jellyfish could be programmed to execute more complex missions, including multiple target depths, extended drift phases, or customized sampling patterns.

The platform (Figure 1) uses a pressure sensor and a closed-loop, state-based algorithm to control swimming behavior, automatically ceasing stimulation at predetermined depths and resuming when the jellyfish returns to the surface. This approach enables the jellyfish to perform repeated vertical-profiling missions while collecting continuous ocean temperature data with a power consumption of 0.10 ± 0.02 W kg^−1^. The balance between jellyfish swimming capability and overall payload buoyancy proved critical for establishing reliable profiling behavior.

The present control strategy is limited to vertical profiling through pressure-triggered stimulation and positive-buoyancy return to the surface. In addition, the state-based control logic can be readily modified for different vertical mission profiles, including alternate target depths, repeated sampling over specified depth intervals, or depth-holding behavior in which stimulation is adjusted to maintain the platform within a programmed depth range. Potential real-world failure modes include loss of stimulation or inconsistent swimming response if an electrode becomes dislodged, and disruption of the intended buoyancy balance. Buoyancy balance is critical because insufficient positive buoyancy could prevent passive return to the surface, whereas excessive positive buoyancy could prevent the jellyfish from overcoming the upward force during stimulated descent. This balance may be affected by salinity differences between laboratory buoyancy tuning and deployment conditions or by excessive bubble formation on the payload surface. When properly balanced, the positive-buoyancy design provides a passive fail-safe for power or stimulation loss by promoting return to the surface, provided the payload remains intact and sufficiently buoyant.

### 3.2. Laboratory Vertical-Profiling Performance

**Figure 2:**
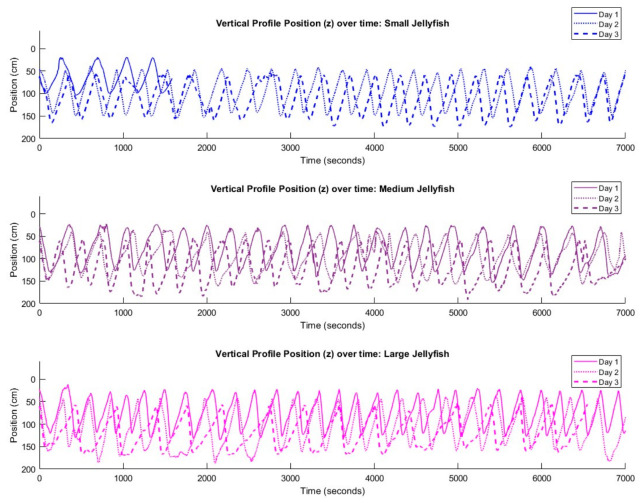
Vertical swimming profiles of biohybrid robot jellyfish of different sizes over time. Vertical position (z) of small, medium, and large *Aurelia aurita* jellyfish recorded during 2 h laboratory swimming trials across three experimental days (solid line = day 1, dotted line = day 2, dashed line = day 3). Each jellyfish was programmed to swim downward to approximately 1.5 m depth before passive ascent. A total of 190 vertical profiles were recorded (n = 53, 67, and 70 profiles for small, medium, and large jellyfish). The cyclic patterns reveal consistent swimming behavior with jellyfish actively descending and passively floating back to the surface throughout the 7000 s observation period (The data for days 2 and 3 were vertically offset by 20 and 40 cm respectively to prevent visual overlap, allowing clearer visualization of swimming patterns across multiple experimental days).

Laboratory experiments with the biohybrid platform demonstrated consistent and sustained vertical-profiling behavior across multiple jellyfish and testing days, validating the robustness of this approach (Figure 2). Each individual (n = 3) completed approximately 20–30 profiles throughout two-hour testing periods in a 2 m tall seawater tank (see the Materials and Methods Section). An outlier was the smallest jellyfish on the first day, which did not maintain swimming activity for the full 2 h duration (Figure 2). During subsequent tests, all other individuals successfully completed the entire 2 h swim protocol. The depth–time profiles shown in Figure 2 reveal regular oscillatory patterns with active descent phases during stimulated swimming followed by passive ascent phases driven by positive buoyancy.

### 3.3. Swimming Velocity and Body Size

**Figure 3:**
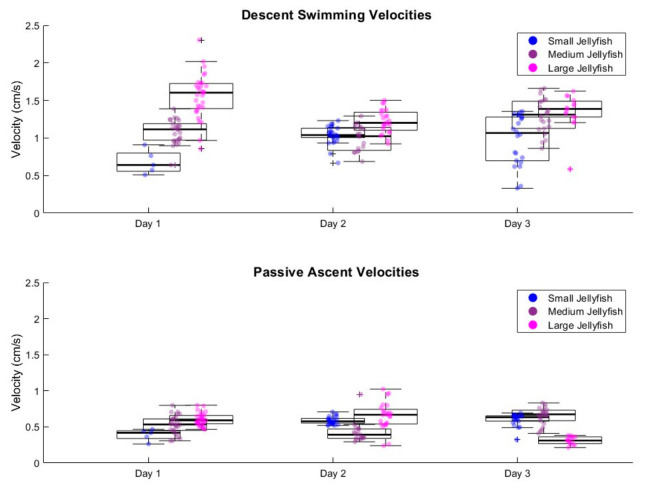
Biohybrid robot jellyfish descent and ascent laboratory swimming trials. Box plots showing swimming speeds of small, medium, and large biohybrid robot jellyfish across three experimental days. Descent velocities demonstrated size-dependent differences, with small jellyfish exhibiting the slowest average descent rates (0.98 ± 0.25 cm/s across all days, n = 53), medium jellyfish showing intermediate average velocities (1.13 ± 0.22 cm/s, n = 67), and large jellyfish achieving the fastest average descent speeds (1.40 ± 0.29 cm/s, n = 70). Statistical analysis revealed significant differences between all size groups during descent (ANOVA: F(2,187) = 42.70, *p* < 0.001). In contrast, the ascent velocities showed no significant size-dependent differences (ANOVA: F(2,187) = 0.82, *p* = 0.44). However, day-to-day variability was observed within each size group, likely reflecting natural biological variation in pulse rate.

There was a significant correlation between jellyfish body size and descent swimming speed, demonstrating that vertical-profiling capabilities can be predictably scaled based on size (Figure 3). This result is consistent with previous observations of natural jellyfish locomotion [36,39]. Statistical analysis confirmed significant differences in descent velocities between size groups (ANOVA: F(2,187) = 42.70, *p* < 0.001). Post hoc comparisons revealed significant differences between all pairwise combinations: small (15.2 cm) vs. medium (17.8 cm)-sized jellies (*p* = 0.0041), small vs. large (18.5 cm) jellies (*p* < 0.001), and medium vs. large jellies (*p* < 0.001). As shown in Figure 3, downward swimming speed scaled positively with body size, whereas the ascent velocities showed no significant differences with body size. This consistency in ascent speeds confirms that the ascent phase is primarily governed by the platform’s positive buoyancy rather than jellyfish size. Calculations indicate that hydrodynamic drag forces during ascent are less than 0.7% of the buoyant force across all specimen sizes, explaining why size differences did not significantly affect ascent velocities (see the calculation in Appendix A).

Day-to-day variability in swimming performance (Table 1; Figure 3) was observed across all individuals, suggesting natural biological variation, which could be influenced by metabolic state or individual responsiveness to electrical stimulation [37,38]. To quantify this variability, coefficients of variation (CVs) were calculated for each individual and testing day using the profile-level velocity distributions summarized in Table 1. Descent CVs ranged from 11.5 to 33.7%, while ascent CVs ranged from 15.8 to 33.3%, indicating moderate profile-to-profile variability across trials. This variability was most likely driven by the endogenous (natural) pulsing of the jellyfish. Although all individuals were tested at approximately the same time each day and experienced the same acclimatization protocol prior to testing (see the Materials and Methods Section), some days exhibited higher endogenous pulsing. During the descent phase, this was sometimes observed as a “double pulse,” where the jellyfish initiated a natural pulse which was quickly followed by the microcontroller’s external signal, prompting another pulse. This double pulse did not allow for the full expansion and relaxation of the jellyfish’s bell, leading to a less efficient pulse. During the ascent phase, variability was more apparent since jellyfish were not programmed to swim during this time. Sometimes jellyfish pulsed intermittently during ascent, while other times pulsing was infrequent or entirely absent. Despite this, differences in ascent velocities remained small, with a difference in average speeds ranging between 0.01 and 0.04 cm s^−1^. Despite these variations, the overall pattern of vertical-profiling speed remained fairly consistent across all individuals and testing days, with larger jellyfish consistently showing faster descent velocities compared to smaller individuals. Despite this moderate variability, larger jellyfish still exhibited higher pooled mean descent velocities, and descent velocity differed significantly among size groups.

### 3.4. Field Testing and Environmental Monitoring

**Figure 4:**
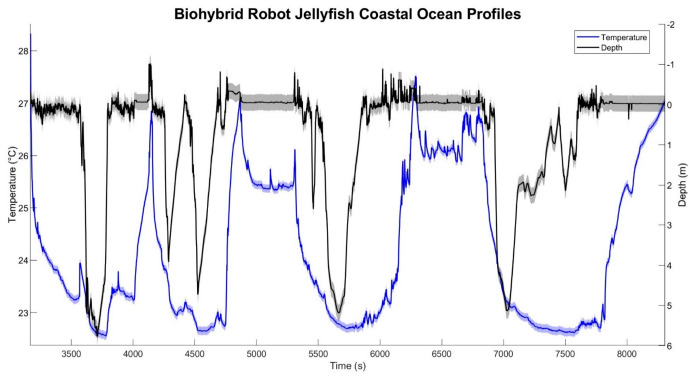
Temperature and depth data collected during biohybrid jellyfish vertical profiling in coastal ocean at Woods Hole Waterfront Park, MA, USA. The depth–time line (black) shows the repeated descent (swim down) and ascent (float up) cycles of biohybrid robot jellyfish vertically profiling in a coastal ocean environment (~6 m depth) while collecting ocean temperature (blue). Temperature is seen to generally decrease with depth and deviations from this trend likely result from lateral drift during profiling through thermally heterogeneous waters.

Field experiments were conducted at two distinct marine locations to test the vertical-profiling capabilities of the biohybrid platform in real-world ocean conditions. Initial testing at the Marine Biological Laboratories (MBL) validated the system functionality in coastal waters, where biohybrid jellyfish completed multiple vertical profiles while collecting temperature data (Figure 4). The temperature measurements acquired during several distinct vertical profiles show spatiotemporal variability in the temperature profile at the site with profile-to-profile variations attributable to sampling in different locations and horizontal drift while sampling. The platform experienced lateral drift across the bay (drifting between an estimated 28–96 m depending on the individual dive), leading to greater temperature heterogeneity as they traversed different water masses. Despite challenging ocean conditions, including wind speeds of 9.0 m s^−1^ and wave heights of 0.67 m, the biohybrid platform profiled in a coastal ocean environment, demonstrating operational resilience beyond controlled laboratory settings.

**Figure 5:**
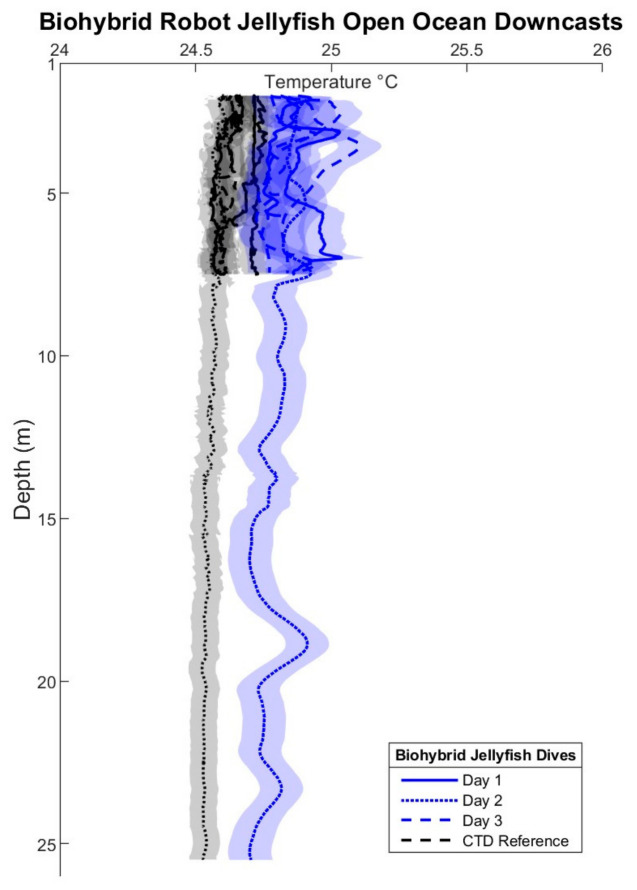
Temperature–depth profiles from biohybrid jellyfish deployments in Florida Keys with reference CTD. Profiles showing temperature measurements collected by the biohybrid jellyfish platform across three testing days (blue lines with shaded uncertainty bands) compared to CTD reference data (black line with gray uncertainty band). The jellyfish platform performed several 6 m downcasts and one 27 m downcast. Minor temperature offsets (<0.5 °C) between jellyfish and CTD measurements may result from the spatial separation (~9 m) from CTD and jellyfish, sensor calibration differences, or biological insulation. The propagated nominal uncertainty for the biohybrid platform–CTD temperature comparison is approximately ±0.1 °C.

Subsequent field testing at Keys Marine Laboratory (KML) in Florida expanded the operational envelope of the system to greater depths and provided a field assessment of sensor performance under natural ocean conditions (Figure 5). Biohybrid jellyfish successfully performed vertical profiles at both a shallow 6 m site and a deeper-water location (27 m) in the open ocean. To assess sensor performance, one diver carried a handheld conductivity–temperature–depth (CTD) sensor (CastAway) as a control reference, enabling comparison to the biohybrid platform’s onboard sensor measurements. The TMP119 sensor has a manufacturer-reported resolution of 0.0078 °C and accuracy of ±0.08 °C, while the CastAway-CTD temperature accuracy is ±0.05 °C. Assuming independent sensor uncertainties, the combined nominal uncertainty is approximately ±0.1 °C, calculated as $(0.08^{2}+0.05^{2}{)}^{1/2}$. These comparisons revealed general agreement in both pressure and temperature measurements, with slight but consistent offsets in temperature and pressure profiles (Figure 5). In the shallow deployment, the jellyfish and CTD profiles showed similar vertical structures, but the biohybrid platform consistently measured temperatures approximately 0.2 °C warmer. The deeper profiles showed more pronounced offsets of approximately 0.3–0.4 °C, likely influenced by the increased separation distance (~9 m) between the jellyfish and diver carrying the CTD. Because the observed 0.2–0.4 °C temperature offset exceeds the expected combined sensor uncertainty, the offset likely reflects deployment-specific factors including spatial separation, sensor encapsulation and placement, partial insulation from jellyfish tissue and waterproofing epoxy, and natural thermal heterogeneity in the water column. However, the system demonstrated good repeatability across multiple specimens, with three different jellyfish exhibiting comparable measurement patterns relative to the reference CTD during similar dive profiles (Figure 5). Future work should include co-located reference CTD comparisons of the fully integrated payload to determine whether a systematic correction factor is needed before operational temperature profiling.

### 3.5. Energy Consumption

Energy consumption measurements across three stimulation trials revealed voltage drops of 4 mV, 4 mV, and 3 mV over approximately 183 s, corresponding to charge removals of 0.83, 0.83, and 0.63 mAh, respectively. The mean power during active swimming was 0.06 ± 0.01 W, yielding a payload mass-normalized consumption of 0.48 ± 0.07 W kg^−1^. With powered descent occupying ~20% of each vertical profile, the cycle-averaged electrical demand was 0.012 ± 0.002 W (0.10 ± 0.02 W kg^−1^). This low power consumption enables extended deployments, with the 250 mAh (3.7 V) battery currently supporting operation for ~3.2 days. Thus, the current ~3.2-day estimate represents operation of the present prototype under the measured stimulation and logging conditions; real-world deployment duration will vary with programmed profile depth, duty cycle, sensor sampling rate, ambient conditions, and future telemetry requirements.

## 4. Conclusions

These results confirm the viability of biohybrid jellyfish as buoyancy-controlled vertical-profiling systems capable of collecting oceanographic data with minimal power requirements (0.10 ± 0.02 W kg^−1^), low cost (<$50 per unit), and a small form factor. The integration of environmental sensors with biohybrid jellyfish leverages the exceptional swimming efficiency of these organisms [33,36], achieving vertical-profiling capabilities with substantially lower energy requirements than many traditional ocean sampling technologies [12,19]. This approach offers a promising pathway toward developing large-scale, energy-efficient networks for oceanographic monitoring to assess climate change impacts, ecosystem dynamics, and ocean circulation patterns [1,2,3].

Future work will build on this first-generation prototype to address key limitations before the platform can function as a long-term ocean monitoring system. Accordingly, the present field trials should be interpreted as demonstrations of autonomous vertical-profiling capability and in situ data collection, rather than as fully operational long-duration ocean-observing deployments. The present platform should therefore be viewed as a first-generation, shallow-depth demonstration of biohybrid vertical-profiling rather than as a replacement for mature deep-ocean platforms such as Argo floats, gliders, or AUVs. Specifically, the current system lacks onboard data transmission, and the field-demonstrated depth capability is currently ~27 m. A critical next step is integrating communications to enable real-time data transmission when the jellyfish surfaces. Further characterization of the temperature sensor offset will also be needed before quantitative operational temperature profiling, including co-located reference CTD comparisons of the fully integrated payload to determine whether a systematic correction factor is needed.

Longer autonomous deployments will also require paired assessment of platform endurance, animal condition, and environmental influences. Although the present battery estimate suggests operation for approximately 3.2 days under measured stimulation and logging conditions, battery life alone does not define operational duration. Prior laboratory work has demonstrated multi-day biohybrid jellyfish swimming and shown that electrically stimulated pulsing affects energetic demand and swimming biomechanics [44]. Future field studies should therefore evaluate how extended stimulation affects feeding capacity, physiological condition, bell morphology, swimming persistence, and post-deployment survival. Environmental deployment factors will also require additional characterization: while jellyfish are naturally resistant to biofouling, the engineered payload is not, and payload fouling could affect buoyancy, drag, or sensor performance. Ocean currents, localization, and recovery logistics will also need to be evaluated for extended field operation.

Depth capability is also presently constrained by the 3D-printed payload rather than the organism. Cost-effective, pressure-tolerant materials could enable substantially deeper operation, for example by selecting pressure-resistant resins such as Formlabs Rigid 10K which is rated for higher-pressure environments (>1000 m). However, achieving increased ocean depth ratings will require pressure testing of the assembled housing geometry, seals, and wall thickness, as well as additional engineering. The present design could also benefit from expanding the sensor suite to include other ocean-relevant parameters such as conductivity and/or dissolved oxygen. Although the current platform cost is low, integrating hardened payloads, satellite telemetry, and additional sensors will increase the overall system cost. To preserve scalability as capabilities expand, future designs could prioritize modular, configurable payloads. With future development, this approach could complement existing oceanographic sampling methods by offering increased mission flexibility through programmable swimming behaviors and enabling distributed observations at scales and in locations that may be difficult for traditional ocean monitoring technologies.

## Figures and Tables

**Table 1:** Vertical swimming performance and repeatability of biohybrid robot jellyfish. Descent and ascent velocities (cm s^−1^; mean ± SD) were measured across multiple testing days for small (15.2 cm diameter), medium (17.8 cm), and large (18.5 cm) biohybrid jellyfish. Coefficients of variation (CVs; %) were calculated as SD/mean × 100 for each individual and testing day to quantify profile-to-profile variability within each 2 h laboratory trial. Statistical analysis revealed significant differences in both descent and ascent velocities across experimental days for all size classes (*p* < 0.05). Despite moderate biological variability, the overall pattern of vertical profiling remained consistent across individuals and days, with larger jellyfish generally showing faster descent velocities than smaller individuals.

Individual Jellyfish	Day	Descent Velocity (cm/s) ± SD	Descent CV (%)	Ascent Velocity (cm/s) ± SD	Ascent CV (%)	ANOVA (Descent)	*p*-Value (Descent)	ANOVA (Ascent)	*p*-Value (Ascent)
**Small**	Day 1	0.68 ± 0.16	23.5	0.57 ± 0.09	15.8	F (2, 50) = 4.89	0.0115	F (2, 50) = 19.98	<0.001
	Day 2	1.04 ± 0.12	11.5	0.54 ± 0.10	18.5		0.0082		
	Day 3	0.98 ± 0.33	33.7	0.56 ± 0.12	21.4		0.0337		
**Medium**	Day 1	1.08 ± 0.16	14.8	0.54 ± 0.16	29.6	F (2, 64) = 14.12	< 0.001	F (2, 64) = 15.28	<0.001
	Day 2	1.00 ± 0.17	17	0.56 ± 0.14	25		< 0.001		
	Day 3	1.30 ± 0.23	17.7	0.58 ± 0.18	31		0.0006		
**Large**	Day 1	1.56 ± 0.30	19.2	0.60 ± 0.20	33.3	F (2, 67) = 13.53	< 0.001	F (2, 67) = 36.49	<0.001
	Day 2	1.22 ± 0.16	13.1	0.58 ± 0.18	31		< 0.001		
	Day 3	1.34 ± 0.25	18.7	0.59 ± 0.19	32.2		0.0173		

## Data Availability

The datasets generated and analyzed in this study are available from the corresponding author upon request.

## Supplementary Materials

The following supporting information can be downloaded at: https://www.mdpi.com/article/10.3390/biomimetics11050325/s1. Table S1. Estimated per-platform component cost for the biohybrid jellyfish payload. Costs represent the estimated consumed component cost for one biohybrid jellyfish payload. Bulk materials, including 3D-printed resin, O-rings, epoxy, O-ring grease, wire, electrode material, connectors, capacitors, and miscellaneous hardware, were prorated based on the approximate amount used per platform. Reusable tools and one-time fabrication supplies were excluded from the estimate. Component prices reflect approximate purchase records or estimated material use at the time of prototype fabrication (2024) and may vary with supplier, purchase date, and quantity. Video S1. Laboratory vertical-profiling experiments showing a biohybrid robotic jellyfish completing repeated vertical profiles in a test tank. The jellyfish was electrically stimulated to swim downward to a depth of approximately 1.5 m, after which stimulation stopped and it floated passively back to the surface. Pulsing during ascent reflects the jellyfish’s natural swimming behavior rather than applied stimulation. Across 18 h of laboratory testing, the jellyfish completed 190 vertical profiles. A thin fishing line used for emergency retrieval is visible and the jellyfish is manually recentered between select profiles to keep it away from the tank walls during descent. Video S2. Output video at 10× speed from the custom semi-automatic MATLAB tracking algorithm used to quantify jellyfish position during laboratory vertical-profiling experiments. Two-hour swimming videos were down-sampled to every 10th frame, and 30 reference keyframes were manually labeled to establish jellyfish position. Between keyframes, jellyfish position was estimated by linear interpolation and refined using brightness-weighted centroid detection within an adaptive search window (150–250 pixels). Background subtraction, contrast enhancement, and thresholding of bright pixels were used to isolate the jellyfish from the tank background and generate vertical position data. Video S3. Field deployment of the biohybrid robotic jellyfish vertical-profiling platform near Woods Hole, Massachusetts. The jellyfish completed vertical profiles at a coastal site approximately 6 m deep while divers followed and recorded the platform underwater. Because of choppy ocean conditions, both the divers and jellyfish moved laterally while the platform swam through the water column. Video S4. Environmental conditions with wind and wave action during field testing of the biohybrid robotic jellyfish platform in coastal waters near Woods Hole, Massachusetts. The deflected dive flag shows where scuba divers were monitoring the biohybrid jellyfish during profiling.

## Appendix A

Hydrodynamic drag forces during ascent were calculated to quantify their relative contribution compared to buoyant forces across different jellyfish sizes. This calculation is intended as a first-order comparison of passive drag relative to net buoyancy during ascent, not as a complete model of unsteady jellyfish-fluid interactions or vortex dynamics during active swimming. The drag forces were estimated using the standard drag equation:

F_drag_ = ½ρv2C_D_A

where ρ is seawater density (1025 kg m^−3^), v is ascent velocity, C_D_ is the drag coefficient, and A is the area of the jellyfish bell.

The ascent velocities were determined from video analysis as described in Materials and Methods, with mean values of 0.48 ± 0.12 cm s^−1^, 0.52 ± 0.09 cm s^−1^, and 0.51 ± 0.11 cm s^−1^ for small (15.2 cm)-, medium (17.8 cm)-, and large (18.5 cm)-diameter jellyfish, respectively. The projected frontal area was calculated as A = π(d/2)^2^ using the measured bell diameter for each individual.

The drag coefficient (C_D_) was estimated as 1.17 for jellyfish during ascent. During ascent, jellyfish were oriented with their bell facing downward due to the payload configuration, meaning they moved backward relative to their normal swimming orientation. This corresponds to the higher drag coefficient for a hemisphere moving in the backward direction (McHenry & Jed, 2003 [45]). For comparison with buoyant forces, the net positive buoyancy of the biohybrid system (0.061 ± 0.003 N) was used as the primary upward force driving ascent motion. Drag force calculations yielded maximum values of 2.51 × 10^−4^ N, 4.04 × 10^−4^ N, and 4.19 × 10^−4^ N for small, medium, and large jellyfish, respectively. These represent 0.41%, 0.66%, and 0.69% of the total buoyant force, confirming that hydrodynamic drag is negligible compared to buoyancy during ascent across all sizes tested.

## References

[1] Benway H.M., Lorenzoni L., White A.E., Fiedler B., Levine N.M., Nicholson D.P., DeGrandpre M.D., Sosik H.M., Church M.J., O’Brien T.D. (2019). Ocean Time Series Observations of Changing Marine Ecosystems: An Era of Integration, Synthesis, and Societal Applications. Front. Mar. Sci..

[2] Golden J.S., Virdin J., Nowacek D., Halpin P., Bennear L., Patil P.G. (2017). Making sure the blue economy is green. Nat. Ecol. Evol..

[3] Pearlman J., Bushnell M., Coppola L., Karstensen J., Buttigieg P.L., Pearlman F., Simpson P., Barbier M., Muller-Karger F.E., Munoz-Mas C. (2019). Evolving and Sustaining Ocean Best Practices and Standards for the Next Decade. Front. Mar. Sci..

[4] Rayner R., Jolly C., Gouldman C. (2019). Ocean Observing and the Blue Economy. Front. Mar. Sci..

[5] Satterthwaite E.V., Bax N.J., Miloslavich P., Ratnarajah L., Canonico G., Dunn D., Simmons S.E., Carini R.J., Evans K., Allain V. (2021). Establishing the Foundation for the Global Observing System for Marine Life. Front. Mar. Sci..

[6] Abraham J.P., Baringer M., Bindoff N.L., Boyer T., Cheng L.J., Church J.A., Conroy J.L., Domingues C.M., Fasullo J.T., Gilson J. (2013). A review of global ocean temperature observations: Implications for ocean heat content estimates and climate change. Rev. Geophys..

[7] Ardyna M., Arrigo K.R. (2020). Phytoplankton dynamics in a changing Arctic Ocean. Nat. Clim. Change.

[8] Johnson G.C., Lyman J.M. (2020). Warming trends increasingly dominate global ocean. Nat. Clim. Change.

[9] Breier J.A., Jakuba M.V., Saito M.A., Dick G.J., Grim S.L., Chan E.W., McIlvin M.R., Moran D.M., Alanis B.A., Allen A.E. (2020). Revealing ocean-scale biochemical structure with a deep-diving vertical profiling autonomous vehicle. Sci. Robot..

[10] Pinkerton M.H., Boyd P.W., Deppeler S., Hayward A., Höfer J., Moreau S. (2021). Evidence for the Impact of Climate Change on Primary Producers in the Southern Ocean. Front. Ecol. Evol..

[11] Roemmich D., Alford M.H., Claustre H., Johnson K., King B., Moum J., Oke P., Owens W.B., Pouliquen S., Purkey S. (2019). On the Future of Argo: A Global, Full-Depth, Multi-Disciplinary Array. Front. Mar. Sci..

[12] Glenn S., Jones C., Twardowski M., Bowers L., Kerfoot J., Kohut J., Webb D., Schofield O. (2008). Glider observations of sediment resuspension in a Middle Atlantic Bight fall transition storm. Limnol. Oceanogr..

[13] Perry M.J., Sackmann B.S., Eriksen C.C., Lee C.M. (2008). Seaglider observations of blooms and subsurface chlorophyll maxima off the Washington coast. Limnol. Oceanogr..

[14] Webb D.C., Simonetti P.J., Jones C.P. (2001). SLOCUM: An underwater glider propelled by environmental energy. IEEE J. Ocean. Eng..

[15] Fossum T.O., Fragoso G.M., Davies E.J., Ullgren J.E., Mendes R., Johnsen G., Ellingsen I., Eidsvik J., Ludvigsen M., Rajan K. (2019). Toward adaptive robotic sampling of phytoplankton in the coastal ocean. Sci. Robot..

[16] Nicholls K.W., Abrahamsen E.P., Heywood K.J., Stansfield K., Østerhus S. (2008). High-latitude oceanography using the Autosub autonomous underwater vehicle. Limnol. Oceanogr..

[17] Tivey M.A., Johnson H.P., Bradley A., Yoerger D. (1998). Thickness of a submarine lava flow determined from near-bottom magnetic field mapping by autonomous underwater vehicle. Geophys. Res. Lett..

[18] Schmidt H., Bellingham J.G., Johnson M., Herold D., Farmer D.M., Pawlowicz R. Real-time frontal mapping with AUVs in a coastal environment. Proceedings of the Symposium on Autonomous Underwater Vehicle Technology.

[19] Yamahara K.M., Preston C.M., Birch J., Walz K., Marin R., Jensen S., Pargett D., Roman B., Ussler W., Zhang Y. (2019). In Situ Autonomous Acquisition and Preservation of Marine Environmental DNA Using an Autonomous Underwater Vehicle. Front. Mar. Sci..

[20] Bradley A.M., Feezor M.D., Singh H., Sorrell F.Y. (2001). Power systems for autonomous underwater vehicles. IEEE J. Ocean. Eng..

[21] Hobson B.W., Bellingham J.G., Kieft B., McEwen R., Godin M., Zhang Y. (2012). Tethys-class long range AUVs—Extending the endurance of propeller-driven cruising AUVs from days to weeks. 2012 IEEE/OES Autonomous Underwater Vehicles (AUV), Southampton, UK, 24–27 September 2012.

[22] Roper D.T., Phillips A.B., Harris C.A., Salavasidis G., Pebody M., Templeton R., Amma S.V.S., Smart M., Phail S.M. (2017). Autosub long range 1500: An ultra-endurance AUV with 6000 km range. OCEANS 2017—Aberdeen, Aberdeen, UK, 19–22 June 2017.

[23] Roquet F., Boehme L., Block B., Charrassin J.B., Costa D., Guinet C., Harcourt R.G., Hindell M.A., Hückstädt L.A., McMahon C.R. (2017). Ocean observations using tagged animals. Oceanography.

[24] Charrassin J.B., Hindell M., Rintoul S.R., Roquet F., Sokolov S., Biuw M., Costa D., Boehme L., Lovell P., Coleman R. (2008). Southern Ocean frontal structure and sea-ice formation rates revealed by elephant seals. Proc. Natl. Acad. Sci. USA.

[25] Costa D.P., Huckstadt L.A., Crocker D.E., McDonald B.I., Goebel M.E., Fedak M.A. (2010). Approaches to Studying Climatic Change and its Role on the Habitat Selection of Antarctic Pinnipeds. Integr. Comp. Biol..

[26] Fedak M. (2004). Marine animals as platforms for oceanographic sampling: A “win/win” situation for biology and operational oceanography. Mem. Natl. Inst. Polar Res..

[27] Fedak M.A. (2013). The impact of animal platforms on polar ocean observation. Deep Sea Res. Part II Top. Stud. Oceanogr..

[28] Treasure A.M., Roquet F., Ansorge I.J., Bester M.N., Boehme L., Bornemann H., Charrassin J.B., Chevallier D., Costa D.P., Fedak M.A. (2017). Marine Mammals Exploring the Oceans Pole to Pole: A review of the MEOP consortium. Oceanography.

[29] Mooney T.A., Katija K., Shorter K.A., Hurst T., Fontes J., Afonso P. (2015). ITAG: An eco-sensor for fine-scale behavioral measurements of soft-bodied marine invertebrates. Anim. Biotelem..

[30] Hussey N.E., Kessel S.T., Aarestrup K., Cooke S.J., Cowley P.D., Fisk A.T., Harcourt R.G., Holland K.N., Iverson S.J., Kocik J.F. (2015). Aquatic animal telemetry: A panoramic window into the underwater world. Science.

[31] Boehme L., Lovell P., Biuw M., Roquet F., Nicholson J., Thorpe S.E., Meredith M.P., Fedak M. (2009). Technical Note: Animal-borne CTD-Satellite Relay Data Loggers for real-time oceanographic data collection. Ocean Sci..

[32] Anuszczyk S.R., Yoder N., Costello J.H., Dabiri J.O., Gemmell B.J., Rutledge K.M., Colin S.P. (2025). Increasing the reliability and versality of jellyfish biohybrid vehicles via species selection and Rhopalia removal. Biomimetics.

[33] Xu N.W., Dabiri J.O. (2020). Low-power microelectronics embedded in live jellyfish enhance propulsion. Sci. Adv..

[34] Anuszczyk S.R., Dabiri J.O. (2024). Electromechanical enhancement of live jellyfish for ocean exploration. Bioinspir. Biomim..

[35] Lucas C.H. (2001). Reproduction and life history strategies of the common jellyfish, Aurelia aurita, in relation to its ambient environment. Hydrobiologia.

[36] Gemmell B.J., Colin S.P., Costello J.H., Stewart C.J., Dabiri J.O., Tafti D., Priya S. (2013). Passive energy recapture in jellyfish contributes to propulsive advantage over other metazoans. Proc. Natl. Acad. Sci. USA.

[37] Arai M.N. (1997). A Functional Biology of Scyphozoa.

[38] Gemmell B.J., Costello J.H., Colin S.P., Stewart C.J., Dabiri J.O., Tafti D., Priya S. (2015). Suction-based propulsion as a basis for efficient animal swimming. Nat. Commun..

[39] Costello J.H., Colin S.P., Dabiri J.O., Gemmell B.J., Lucas K.N., Sutherland K.R. (2020). The Hydrodynamics of Jellyfish Swimming. Annu. Rev. Mar. Sci..

[40] Xu N.W., Lenczewska O., Wieten S.E., Federico C.A., Dabiri J.O. (2025). Ethics of biohybrid robotics and invertebrate research: Biohybrid robotic jellyfish as a case study. Bioinspir. Biomim..

[41] Xu N.W., Townsend J.P., Costello J.H., Colin S.P., Gemmell B.J., Dabiri J.O. (2020). Field Testing of Biohybrid Robotic Jellyfish to Demonstrate Enhanced Swimming Speeds. Biomimetics.

[42] Nelson R.F. (2000). Power requirements for batteries in hybrid electric vehicles. J. Power Sources.

[43] Texas Instruments (2013). Battery Fuel Gauges: Theory and Application (SLUA801). https://www.ti.com/lit/pdf/slua801.

[44] Anuszczyk S.R., Phaychanpheng K., Dabiri J.O. (2026). From wake dynamics to energy consumption in free-swimming biohybrid robotic jellyfish: A multiscale analysis. arXiv.

[45] McHenry M.J., Jed J. (2003). The ontogenetic scaling of hydrodynamics and swimming performance in jellyfish (*Aurelia aurita*). J. Exp. Biol..

