# Supplementary material for: Biohybrid Robotic Jellyfish for Swimming-Enhanced Vertical Ocean Profiling
Source: Biomimetics (Basel). 2026 May 7;11(5):325. doi: 10.3390/biomimetics11050325 (PMC13204357; doi:10.3390/biomimetics11050325)
Supplement: Supplementary file 1 [file biomimetics-11-00325-s001.zip › biomimetics-4269766-supplementary.pdf]

Supplementary

Supplementary Table S1. Estimated per-platform component cost for the biohybrid jellyfish payload. Costs represent the estimated consumed component cost for one biohybrid jellyfish payload. Bulk materials, including 3D-printed resin, O-rings, epoxy, O-ring grease, wire, electrode material, connectors, capacitors, and miscellaneous hardware, were prorated based on the approximate amount used per platform. Reusable tools and one-time fabrication supplies were excluded from the estimate. Component prices reflect approximate purchase records or estimated material use at the time of prototype fabrication (2024) and may vary with supplier, purchase date, and quantity.

Component	Estimated per-platform cost
Pressure sensor	$19.00
Temperature sensor	$5.11
Micro processor	$9.95
250 mAh battery	$4.00
microSD card reader	$1.00
3D-printed resin	$5.00
Buck converter	$1.00
O-rings	$0.50
Epoxy / O-ring grease	$0.50
wire	$0.50
rod / electrode material	$1.50
JST connector / capacitor / misc.	$0.75
**Estimated total**	**$48.81**

Supplementary Video S1. Laboratory vertical profiling experiments showing a biohybrid robotic jellyfish completing repeated vertical profiles in a test tank. The jellyfish was electrically stimulated to swim downward to a depth of approximately 1.5 m, after which stimulation stopped and floated passively back to the surface. Pulsing during ascent reflects the jellyfish's natural swimming behavior rather than applied stimulation. Across 18 h of laboratory testing, the jellyfish completed 190 vertical profiles. A thin fishing line used for emergency retrieval is visible and the jellyfish is manually recentered between select profiles to keep it away from the tank walls during descent.

Supplementary Video S2. Output video at 10× speed from the custom semi-automatic MATLAB tracking algorithm used to quantify jellyfish position during laboratory vertical profiling experiments. Two-hour swimming videos were down-sampled to every 10th frame, and 30 reference keyframes were manually labeled to establish jellyfish position. Between keyframes, position was estimated by linear interpolation and refined using brightness-weighted centroid detection within an adaptive search window (150-250 pixels). Background subtraction, contrast enhancement, and thresholding of bright pixels were used to isolate the jellyfish from the tank background and generate vertical position data.

Supplementary Video S3. Field deployment of the biohybrid robotic jellyfish vertical profiling platform near Woods Hole, Massachusetts. The jellyfish completed vertical profiles at a coastal site approximately 6 m deep while divers followed and recorded the platform underwater. Because of choppy ocean conditions, both the divers and jellyfish moved laterally while the platform swam through the water column.

Supplementary Video S4. Environmental conditions with wind and wave action during field testing of the biohybrid robotic jellyfish platform in coastal waters near Woods Hole, Massachusetts. The deflected dive flag shows where scuba divers were monitoring the biohybrid jellyfish during profiling.
